# Nanomedicine for autophagy modulation in cancer therapy: a clinical perspective

**DOI:** 10.1186/s13578-023-00986-9

**Published:** 2023-03-04

**Authors:** Tania B. López-Méndez, Miguel Sánchez-Álvarez, Flavia Trionfetti, José L. Pedraz, Marco Tripodi, Marco Cordani, Raffaele Strippoli, Juan González-Valdivieso

**Affiliations:** 1 NanoBioCel Group, University of the Basque Country (UPV/EHU), Vitoria-Gasteiz, Spain; 2 Biomedical Research Networking Center in Bioengineering, Biomaterials and Nanomedicine (CIBER-BBN), Vitoria-Gasteiz, Spain; 3 Area of Cell and Developmental Biology. Centro Nacional de Investigaciones Cardiovasculares (CNIC), Madrid, Spain; 4 Instituto de Investigaciones Biomédicas Alberto Sols (IIB), Madrid, Spain; 5 Department of Molecular Medicine, Sapienza University of Rome, Rome, Italy; 6 National Institute for Infectious Diseases L. Spallanzani IRCCS, Rome, Italy; 7 Department of Biochemistry and Molecular Biology, School of Biology, Complutense University, Madrid, Spain; 8 Instituto de Investigaciones Sanitarias San Carlos (IdISSC), Madrid, Spain; 9 Department of Radiology, Molecular Imaging Innovations Institute (MI3), Weill Cornell Medicine, New York, USA

**Keywords:** Cancer, Nanomedicine, Biomaterials, Clinical trials, Autophagy

## Abstract

In recent years, progress in nanotechnology provided new tools to treat cancer more effectively. Advances in biomaterials tailored for drug delivery have the potential to overcome the limited selectivity and side effects frequently associated with traditional therapeutic agents. While autophagy is pivotal in determining cell fate and adaptation to different challenges, and despite the fact that it is frequently dysregulated in cancer, antitumor therapeutic strategies leveraging on or targeting this process are scarce. This is due to many reasons, including the very contextual effects of autophagy in cancer, low bioavailability and non-targeted delivery of existing autophagy modulatory compounds. Conjugating the versatile characteristics of nanoparticles with autophagy modulators may render these drugs safer and more effective for cancer treatment. Here, we review current standing questions on the biology of autophagy in tumor progression, and precursory studies and the state-of-the-art in harnessing nanomaterials science to enhance the specificity and therapeutic potential of autophagy modulators.

## Introduction

Autophagy encompasses a number of self-catalytic mechanisms which enable the elimination of macromolecules and organelles, protecting from any potential toxicity upon damage and making available their constituent building blocks for anabolism and energy obtention [1–3]. Its physiological and physio-pathological relevance is difficult to overstate, and the number of roles it plays in development, tissue repair or disease continues to grow [1].

Autophagy plays several roles in tumor biology which depend on different context features. Sustained autophagy at later disease stages can promote cancer cell survival by ensuring the removal of damaged organelles and macromolecules, and the fulfilling of the high metabolic demands of proliferating tumor cells exposed to stressful conditions, such as nutrient deprivation, oxidative stress, hypoxia, or exposure to anti-cancer agents. However, induction of autophagy can also promote cell death, likely through excessive degradation of cellular constituents and organelles [2, 3]. Thus, autophagy can be described as either tumor suppressing or promoting mechanism depending on context; nevertheless, its frequent role in tumor progression, survival to environmental challenges (hypoxia, detachment) or chemoresistance positions autophagy as a priority therapeutic target. For instance, in systems where autophagy acts as a mechanism of survival and chemoresistance, its pharmacological inhibition may trigger apoptosis [1]. Intervention of autophagy may synergize with other chemotherapy strategies simultaneously compromising other mechanisms essential for tumor cell survival, as revealed by combinatorial genetic screens [4, 5].

Despite the antitumor therapeutic potential of autophagy modulators, their use in a clinical setting has been limited so far. Currently available autophagy modulators show poor bioavailability because of low solubility in aqueous media and non-targeted delivery, leading to modest therapeutic efficacy and favoring undesired effects. Novel approaches are thus warranted to harness the potential of these modulators.

Nanotechnology is revolutionizing biomedicine and pharmaceutical industry; this new field is generally referred to as nanomedicine. Several nanoparticles (NPs) have been proposed as drug carriers and imaging tools [6]. Nanoparticles can be custom-designed to exhibit specific physicochemical properties, such as charge, shape, surface decoration and a high surface-to-volume ratio; this makes them particularly attractive as safe carriers of compounds to specific target sites.

Besides acting as nano-carriers, nanoparticles of specific materials may have the *intrinsic* ability of altering the complex network of signaling pathways and molecules involved in autophagy regulation, and thus represent an exciting therapeutic approach against cancer disease. [7].

In this review, we aim to provide an updated picture of the different pharmacological approaches designed so far to treat cancer, focusing on existing clinical trials where different autophagy modulators are carried by NPs, including siRNA cellular nanodelivery and modulation of autophagy by magnetic hyperthermia.

## Autophagy

**Fig. 1:**
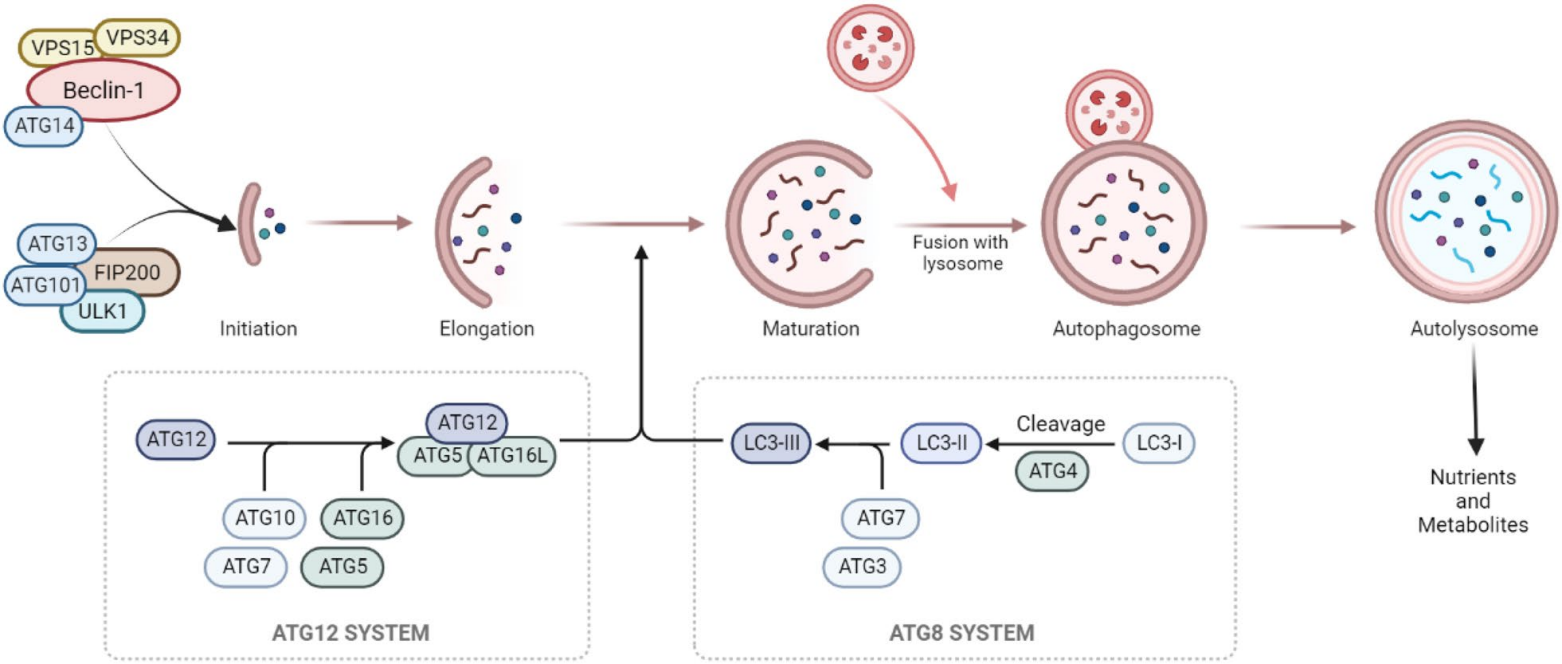
Schematic view of the main phases and molecules implicated in the autolysosome formation during the autophagic process

Macroautophagy—commonly simply referred to as autophagy—is a degradative process by which intracellular macromolecules and organelles are engulfed by autophagic vesicles, delivered to lysosomes, and broken down to their molecular building blocks. Autophagy is a crucial process to ensure cell survival in the face of different sources of stress and damage, such as nutrient starvation [2]. Upon initiation, targeted structures are embedded into double-membrane vesicles—autophagosomes—which fuse with the lysosomal compartment (Fig. 1), allowing for the degradation of its cargo by lysosomal hydrolases [2]. The autophagic machinery requires the activation of autophagy-related genes (ATGs), which are essential for the formation of double-membrane autophagosome vesicles [2]. Vesicular protein sorting 34 (Vps34), a class III PI-3 kinase, interacts with beclin-1 and other autophagy-related proteins and plays a critical role in early vesicle nucleation, with the assistance of ATG2 and ATG9, which provide phospholipids for autophagosome membrane expansion [2, 8]. Autophagosomes are further matured by the ATG14/beclin-1/VPS34 complex. Autophagy initiators unc-51-like kinase 1 (ULK1) and beclin-1 complexes are positively regulated through ubiquitylation by the cofactor AMBRA1 [3, 9]. The mammalian homolog of ATG8, also called LC3B, is expressed as a full-length cytosolic protein that, upon induction of autophagy, is cleaved by ATG4—a cysteine protease—to generate LC3B-I. The carboxy-terminal glycine exposed by ATG4-dependent cleavage is then activated in an ATP-dependent manner by the E1-like ATG7 and transferred to ATG3, to generate the active isoform LC3B-II. Finally, recruitment of LC3B-II into the growing phagophore is dependent on ATG5–ATG12 interaction, which favors LC3B-II binding to both the internal and external surfaces of autophagosomes [3]

Autophagy regulation is intimately linked to energy status and nutrient sensing. AMP-activated protein kinase (AMPK) is a central regulator of autophagy. AMPK-dependent phosphorylation inhibits mTORC1 through TSC2 and Raptor in response to cellular energy deficit, and activates ULK1, which is a required step to trigger the autophagic machinery [10]. In energetic stress situations, ULK1 complex promotes autophagy by targeting several downstream effectors, such as the actin-associated motor protein myosin II and ATG9 [10].

Mechanistic target of rapamycin (mTOR) participates of two distinct functional complexes, mTORC1 and mTORC2 [10]. mTORC1 is a multiprotein complex composed of mTOR, mLST8, and Raptor, sensitive to rapamycin inhibition [11]. mTORC1 is a central integrator of several cues (nutrient sensing, growth factor signaling, stress signaling pathways) to regulate cell growth and survival, proliferation, and the balance between most anabolic processes, including protein synthesis and autophagy [12, 13].

The observation that mTORC1 pharmacological inhibition is sufficient to induce autophagy even in the presence of nutrients, highlights the role of mTORC1 complex as a powerful repressor of autophagy [14]. Genetic and biochemical studies demonstrated that the inhibition of ULK1 by mTORC1-dependent phosphorylation is a crucial point involved in autophagy repression [15]. Indeed, recent studies showed that mTORC1 can phosphorylate ULK1 on Ser757 to favor autophagy blockage [16].

mTORC2 is largely insensitive to rapamycin [17], and includes as specific components Rictor and SIN1. Additionally, Protor-1/2 and Deptor can bind to mTORC2 [18]. mTORC2 participate of chaperone-mediated autophagy [19] and may have a role in autophagy via FoxO3 [11]. mTORC2 also positively regulates protein kinase B (PKB/AKT) by phosphorylating its Ser 473 residue; this node also feeds information from growth factor signaling onto mTORC1, and could thus further contribute to the attenuation of autophagy through ULK1 phosphorylation [20].

## Autophagy in cancer

Autophagy has been involved in physiological processes including normal development, and in a variety of human diseases, including cancer, neurodegenerative diseases and muscular disorders [21]. Growth signaling, nutrient demand and availability and the balance between anabolic pathways and energy status are all almost invariably altered in tumor cells; accordingly, autophagy fluxes are frequently altered in cancer disease [21]. Autophagy can both promote cancer cell death or survival and may be considered either driver or consequence of tumorigenesis. This conundrum is derived from context-dependent conditionals such as tumor type and stage, microenvironment context (i.e., nutrient availability, hypoxia) and cell intrinsic properties [21].

Constitutive autophagy may have a protective role in tumor cells by removing damaged organelles or recycling misfolded macromolecules [22]. Moreover, autophagy fulfills the high metabolic demands of proliferating tumor cells exposed to stressful conditions, such as nutrient deprivation, oxidative stress, hypoxia, or exposure to anti-cancer therapies [23]. Hypoxic microenvironments trigger HIF-1α-dependent and -independent autophagy, which also contributes to tumor survival [24]. Interestingly, cancers harboring activating KRAS mutations have a high basal rate of autophagy, even in conditions of active proliferation [25]. Studies based on pancreatic cancer xenografts and genetically transformed murine models support that pharmacological and genetic inhibition of autophagy may result in tumor regression [21]. Thus, autophagy appears to serve as a pro-survival mechanism for tumor cells by enhancing stress tolerance and providing an alternative nutrient source by which cancer cells can meet their abnormally high nutrient and energy demands.

However, a large body of evidence also suggests that induction of autophagy in tumor cells can promote cell death—cell death-type II—through enhanced degradation of cellular components and organelles. Autophagy has thus been considered tumor-suppressive in several experimental systems. In fact, tumor cells frequently exhibit driving features, such as increased ROS production and induction of genetic mutations, that imply an attenuation of excessive autophagy [26]. Impaired autophagy may favor the accumulation of damaged mitochondria that are a potential source of ROS, leading to genetic mutations and tumor progression [27]. Defects in autophagy may favor DNA damage, aneuploidy, and genomic instability, promoting tumor transformation [28], and a chronic inflammatory state that may further favor tumorigenesis through inflammatory cytokine and chemokine production [26]. Thus, impaired autophagy can support tumor progression by promoting both genomic instability and inflammation. In support of the hypothesis of a tumor-suppressive autophagy, mice with heterozygous deletions of beclin-1 develop spontaneous tumors. Allelic loss of beclin1 was also observed in 40–75% of breast, ovarian, and prostate cancers [29]. Moreover, increased expression of an autophagy adaptor protein, p62/SQSTM1, due to autophagy inhibition, promoted tumor progression through several mechanisms [30]. Opposite to their wild-type counterpart, mutant p53 proteins can promote tumorigenesis favoring the acquisition of DNA mutations, leading to a reduced response to chemotherapy and to a more severe prognosis in cancer patients [31]. The mechanism of autophagy inhibition by gain of function p53 proteins may reside in mTOR stimulation [32–34] and AMPK inhibition [35, 36]. This acquisition of pro-oncogenic functions by mutant p53 is an essential turning point in tumorigenesis. On the other hand, autophagy can promote the degradation of mutant p53 proteins in a functional interplay that may be central in tumor pathogenesis and in the response to antitumor therapies [37].

## Autophagy modulating drugs

Autophagy is often associated with tumor survival and chemoresistance [1, 2, 22]. Autophagy inhibition can thus sensitize chemo-resistant cells to chemotherapeutics, favoring tumor apoptosis [38]. For example, genetic depletion of Atg5, Atg7 or beclin1 may rescue tamoxifen resistance in HER-positive breast cancer cells [5]. The autophagy inhibitor 3-methyl-adenine (3-MA) increases the antitumor effect of trastuzumab (Tmab), an HER2-specific monoclonal antibody [5]. Resistance to cisplatin in ovarian cancer is often associated increased autophagy, and Atg5 genetic deletion in these cells can induce apoptosis [39].

The only compounds intervening autophagy currently approved by FDA for clinical use are the antimalarial drug chloroquine (CQ) and its derivative, hydroxychloroquine (HCQ) [38]. HCQ is a lysosomotropic agent reported to be able to inhibit lysosomal acidification thus inhibiting the autophagic flux [40, 41].

Of note, CQ activity is strongly diminished in conditions of acidic pH; this may explain the limited effect exhibited by CQ in vivo, since the tumor microenvironment is frequently acidic due to enhanced glycolytic rates and tissue damage [42].

HCQ enhanced the activity of erlotinib, an EGFR inihibitor, in a phase I study conducted in advanced non-small-cell lung cancer patients [43]. In renal carcinoma cell lines, HCQ enhanced the activity of mTOR inhibitors, such as everolimus, inhibiting mitochondrial oxygen consumption and promoting apoptosis through inhibition of S6 phosphorylation [44]. In estrogen receptor-positive (ER +) breast cancer cell lines, the combination of HCQ and tamoxifen (TAM) was more effective in increasing the responsiveness to the anti-estrogen therapy, than monotherapy [45].

However, exacerbated autophagy induction upon cytotoxic drug treatment or by direct autophagy induction, can also lead to tumor cell death. For example, temozolomide, an alkylating agent with a pro-autophagy activity, displays synergistic induction of apoptosis in glioblastoma with dasatinib, a tyrosine kinase inhibitor [46]. Histone deacetylase (HDAC) inhibitors [47] and proteasome inhibitors (PI) may also act as autophagy inducers: Bortezomib, a PI used in the treatment of hematological malignancies, increases early formation of autophagosomes and LC3-II expression in prostate cancer cells [48]. A well-known class of autophagy inducers including the mTOR inhibitors temsirolimus and everolimus, have been studied in phase III trials and found effective in therapy of advanced renal cell carcinomas [49]. Everolimus has been approved by the FDA, as an antiangiogenic compound, in renal cell carcinoma, advanced breast cancer, and pancreatic neuroendocrine tumors [49]. Despite this, in clinical trials anticancer treatments based exclusively on mTOR inhibition have demonstrated high resistance rates [50]. Numerous phase I/II clinical trials have investigated the combination of HCQ with mTOR inhibitors in renal cancer, multiple myeloma and advanced solid tumors, as reviewed by Duffy et al. [51].

The relationship between cancer metabolism and autophagy is being evaluated in a clinical trial (NCT01206530) combining HCQ with chemotherapy in patients with advanced colorectal cancer [52]. Another trial (NCT02042989) aims to correlate the effects of combined proteasome and HDAC inhibition on autophagy in patients with advanced p53 mutant malignancies using vorinostat, a broad spectrum HDAC inhibitor, and the proteasome inhibitor MLN9708 [53]. HDAC family members and other epigenetic regulators control autophagy induction through several mechanisms, including regulation of autophagic machinery core gene expression [54]. The increased activity of autophagy after treatment with HDAC inhibitors significantly blunts HDAC inhibitor anticancer activity [54]. The induction of autophagy also occurs in response to proteasome inhibitors, and is believed to play a role in cancer resistance [55]. This sets the basis for early-phase and clinical trials for combined therapies inhibiting autophagy and HDAC [56] or proteasome [57].

The growing number of ongoing trials reflects the relevance that autophagy modulation can have in combinatorial treatments to overcome the existing resistance in some cancer therapies. Recent studies have highlighted that autophagy, besides leading to severe metabolic changes and chemoresistance, also plays an immunomodulatory role and may be exploited to enhance tumor immunotherapy [58].

## Nanocarriers for drug delivery in autophagy modulation

**Fig. 2:**
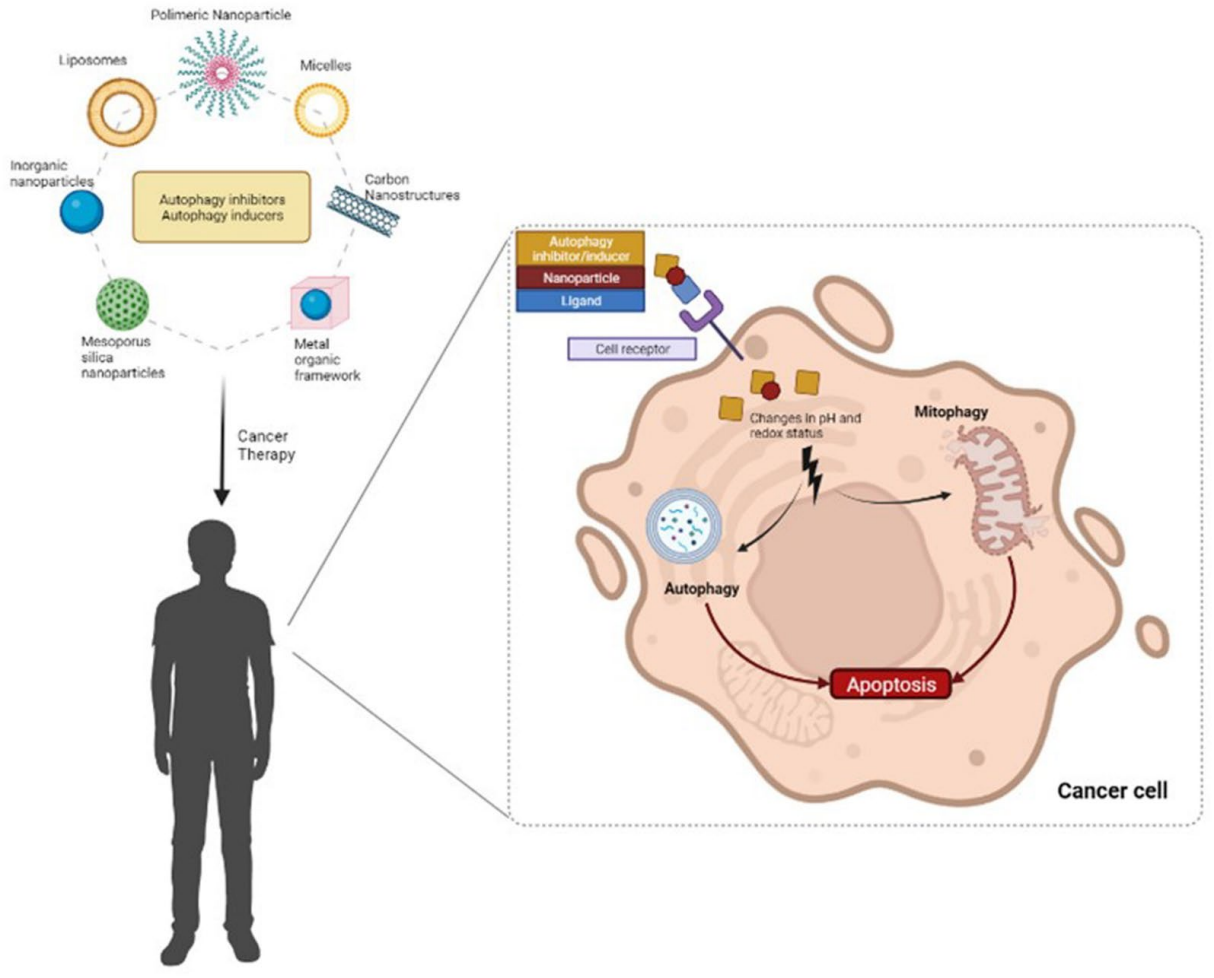
Nanomedicine approaches in drug delivery modulating autophagy in cancer

Several hurdles hamper the use of currently available drugs intervening autophagy in clinical settings. Autophagy modulators often exhibit low bioavailability due to both low solubility in aqueous media and non-targeted delivery, as well as reduced activity in an acidic microenvironment [42]. Novel strategies are thus warranted to leverage on autophagy intervention (Fig. 2).

Recently, nanotechnology-based therapeutic solutions have been developed, generally referred to as nanomedicine [59]. Several nanoparticles (NPs) have been designed to act as both drug carriers and imaging tools [6]. NPs are endowed with specific physicochemical properties, such as charge, shape, surface decoration and a high surface-to-volume ratio, that theoretically make them particularly attractive to load and deliver small compounds to specific targets [60].

Nanomedicine may favor the preferential accumulation of systemically administered chemotherapeutics at tumor sites via the enhanced permeability and retention effect (EPR). In this case, the leaky vascular system and reduced lymphatic drainage characteristic of the tumor microenvironment increases the specificity of the therapeutic system in in vivo settings [61].

Extensive research focusing on cancer nanomedicine has generated nanostructures capable of overcoming biological barriers and transport chemotherapeutic agents to targeted sites while minimizing harmful effects on healthy tissues [6] Moreover, the surface of NPs can be chemically modified by conjugating functional moieties, such as nucleic acids and targeting ligands, in order to increase targeted delivery to tumor sites, maximizing chemotherapy efficacy [6].

Overall, nanomaterials have been explored as potent modulators of autophagy through several mechanisms, and are being actively studied as new therapeutic tools against cancer [7]. A list of nanocarriers described according to their autophagy-related targets is shown in Table 1.

**Table 1:** List of nanocarriers described in this review according to the targets related to autophagy

Target	Therapeutic molecule	Combined molecule	Nanosystem	Disease	References
Autophagosome fusion with lysosomes	Chloroquine	Cisplatin	Hybrid dendritic–linear–dendritic block copolymers (HDLDBCs)	Cervical cancer and lung adenocarcinoma	[62]
Lysosomes	Lys-05	Lysosomotropic detergent (MSDH)	Isaminoquinoline nanoparticle	Pancreatic cancer	[64]
	N-((2-hydroxy-3-trimethylammonium) propyl) chitosan chloride (HTCC)		Fe3O4 magnetic NPs	Gastric carcinoma	[75]
mTOR	AZD8055		Albumin nanoparticles	Uveal melanoma	[76]
	Rapamycin		Albumin nanoparticles	Multiple myeloma	[82]
	Rapamycin		Albumin nanoparticles	Breast cancer	[83]
	Rapamycin		Albumin nanoparticles	Untreatable advanced nonhematologic malignancies	[84]
	Rapamycin		Albumin nanoparticles	Non-hematologic malignancies	[85, 86]
	Rapamycin		Albumin nanoparticles	Perivascular epithelioid cell tumors (PEComa) and patients with a malignancy with relevant genetic mutations or mTOR pathway activation	[87]
	Rapamycin	Pazopanib	Albumin nanoparticles	Non-adipocytic soft tissue sarcomas	[88]
	Rapamycin	Nivolumab	Albumin nanoparticles	Non-hematologic malignancies	[89]
	Rapamycin	FOLFOX, Bevacizumab	Albumin nanoparticles	Metastatic colorectal cancer	[90]
	Rapamycin	Temozolomide and irinotecan	Albumin nanoparticles	Recurrent or refractory solid tumors	[91]
	Rapamycin	Gemcitabine	Albumin nanoparticles	Non-muscle invasive bladder cancer	[92]
	Paclitaxel		Albumin nanoparticles	Metastatic breast cancer	[93]
	Paclitaxel	Gemcitabine	Albumin nanoparticles	Pancreatic cancer	[94]
	Paclitaxel	Carboplatin	Albumin nanoparticles	Non-small-cell lung cancer	[95]
	Rapamycin		Albumin nanoparticles	Advanced solid tumors	[96]
	Rapamycin		Albumin nanoparticles	Solid tumors	[97]
	Everolimus		Albumin nanoparticles	Metastatic melanoma	[98]
	Paclitaxel	Rapamycin	Albumin nanoparticles	Advanced solid tumors	[99]

Treatment with cisplatin and chloroquine in micelles formed by self-assembling hybrid dendritic-linear-dendritic block copolymers (HDLDBCs) increased cytotoxicity in tumor cells, while maintaining a limited cytotoxic activity in non-transformed cells [62]. Lys-05, a potent autophagy inhibitor that deacidifies the lysosome [63], was let to interact with a lysosomotropic detergent (MSDH). The resulting single-drug nanoparticles exhibited excellent pharmacokinetic and toxicological profiles and were more efficacious against tumors in vivo [64]. The surface of gold nanoparticles (Au-NPs) can be easily functionalized with chemotherapeutics, antibodies, or nucleic acids, such as snake-venom-protein toxin NKCT1, anti- epidermal growth factor receptor (EGFR) monoclonal antibody, trastuzumab, or quercetin, making them excellent autophagy inducers for cancer therapy [65, 66].

In addition to their cargo activity, some nanoparticles have the intrinsic ability of regulating various stages of autophagy, thus constituting an additional pharmacological tool in tumor therapy [7]. Metal-based NPs, such as bare iron-oxide NPs (IO-NPs), cuprous (Cu-NPs) and copper oxide nanoparticles (CO-NPs), may induce autophagy through several mechanisms, including oxidative stress, dysregulation of Akt/AMPK/mTOR pathway, block of phagolysosome formation, mitochondrial damage, and ER stress in a time- and dose-dependent manner exhibiting a significant cytotoxicity in lung, breast and cervix cancer cells, but not in normal cells [67–69]. Besides displaying an ability to induce autophagy, these nanomaterials can act as nanocarriers and deliver specific biomolecules intervening autophagy in cancer cells. For example, IO-NPs when conjugated to anti-EGFR monoclonal antibodies suppressed lung tumor growth both in vitro and in vivo, by abrogating G2/M cell-cycle arrest and inducing DNA damage, autophagy, and apoptosis [70]. In another study, it has been shown that the photothermal effect induced by IO-NPs (see below) could induce protective autophagy in a laser dose-dependent manner in breast cancer cells in vitro and in vivo, and the inhibition of autophagy would enhance the photothermal cell killing by increasing cell apoptosis. Therefore, the combination of drugs intervening autophagy and photothermal agents may represent a novel therapeutic approach [71]. Chiral nanomaterials are frequently used to interfere with the autophagic machinery in cancers [72], and chiral polymer-modified nanostructures may counteract tumor progression i*n vivo* [73]. D- and L-cysteine-modified Cu2 − xS nanoparticles (NCs) have been reported to lead to ROS accumulation in cancer cells, promoting autophagy [74].

Novel chitosan chloride (HTCC)/alginate-encapsulated Fe_3_O_4_ NPs (HTCC–MNPs) increase autophagy through the co-localization of LC3 with the lysosomes, inducing cytotoxicity in gastric carcinoma cells rather than not tumoral cell line [75].

Albumin-based nanoparticles, such as albumin-stabilized gold nanoclusters (ABN), show interesting features in biological environments—including in vivo settings—such as low toxicity and remarkable stability. These nanomedicines have been recently exploited for autophagy induction by delivering AZD8055 (ABN-AZD), a powerful mTOR kinase inhibitor, for the treatment of uveal melanoma [76]. Notably, the drug has been linked to ABN using a disulfide moiety, instructing its release specifically inside of tumoral cells, in presence of high amount of glutathione.

Hence, many nanostructures may acquire exceptional medical and toxicological relevance due to their inherent chemical activity within the cellular environment, or by delivering drugs and bioactive compounds active in autophagosome formation or in the related signaling pathways. Hence, autophagy targeting with innovative tools, as nanostructure-based strategies, is acquiring clinical importance as a synergist approach for cancer therapy.

## Clinical trials with nanocarriers involving autophagy modulation

As mentioned above, PI3K/AKT/mTOR pathway inhibitors (i.e. rapamycin, everolimus) are well-known autophagy inductors [77–79] and their combination with other cytotoxic molecules could enhance antitumor efficacy and restrict repopulation of tumor cells between cycles of cytotoxic drugs. Mondesire et al. [80] showed synergy between paclitaxel, an anticancer drug that induces microtubule assembly and stabilization with effectiveness in solid tumors [81], and mTOR inhibitor rapamycin, with enhanced paclitaxel-induced apoptosis.

Nanoparticles of albumin–bound rapamycin (nab-rapamycin; also referred as ABI-009) make unnecessary the use of toxic solvents due to their ability to bind hydrophobic drugs to albumin. In preclinical studies these nanocomplexes were non-toxic and very efficient in different cancer types since they moderated cell viability and hamper mTOR downstream signaling in several xenograft tumor models, including multiple myeloma and breast tumor [82–84]. Furthermore, in human cancer xenograft models, nab-rapamycin alone is able to counteract cancer growth for approximatively 75% and its anticancer effect was increased by combining autophagy inducers [84]. A List of clinical trials testing the efficacy of drugs active in autophagy modulation in cancer and pulmonary/liver fibrosis is shown in Table 2, whereas a list of preclinical studies and clinical trials testing the efficacy of magnetic nanoparticles in targeting solid tumors is shown in Table 3. Phase I studies (NCT00635284, NCT02646319, NCT03817515) [85–87] showed that clinical dose of ABI-009 complexes (100 mg/m^2^ weekly, for 4 weeks) did not show any toxicity and strongly inhibited mTOR targets S6K and 4EBP1 with initial proofs of response and disease stabilization in patients with unresectable and metastatic solid malignancies [84]. Actually, several clinical trials in phase I/II are ongoing to investigate the maximum tolerated dose (MTD), disease control rate (DCR) and progression free survival (PFS) resulting from the combination of ABI-009 nanocomplex with pazopanib (NCT03660930) [88], nivolumab (NCT03190174) [89], FOLFOX, Bevacizumab (NCT03439462) [90], temozolomide and irinotecan (NCT02975882) [91] in patients with advanced sarcomas, metastatic colorectal cancer and in pediatric patients with refractory primary central nervous system cancers.

**Table 2:** List of clinical trials testing the efficacy of drugs active in autophagy modulation in cancer and pulmonary/liver fibrosis

Therapeutic name	Mechanism of action	Delivery system	Target(s)	Cancer type	Phase	NCT ID
Chloroquine (CQ)	Inhibits autophagosome fusion with lysosome and autophagosome degradation	Hybrid dendritic-linear-dendritic block copolymers	Lysosomotropic agent; lysosomal pH	Non-small-cell lung cancer	I/II	NCT00969306
Hydroxychloroquine (HCQ)	Inhibits lysosomal acidification and prevent the degradation of autophagosomes	Liposomes decorated with a pH-sensitive TH-RGD targeting peptide	Lysosomotropic agent; lysosomal pH	Prostate cancer	II	NCT00726596
				Breast cancer	II	NCT04841148
				Solid tumors undergoing radiation therapy for bone metastases	I	NCT01417403
HCQ + Tamoxifen (TAM)	Antiestrogenic activity		Lysosomotropic agent; lysosomal pH; estrogen receptor	Breast	I/II	NCT01023477
Temozolomide	Induces G2 cell cycle arrest	ABI-009 nanocomplex	Genotoxic stress	Refractory solid tumors	I	NCT02975882
Dasatinib	ATP-competitive small-molecule inhibitor		BCR/ABL and Src family tyrosine kinases	Glioblastoma	II	NCT00423735
Bortezomib	Increases the early formation of autophagosomes and LC3-II	Liposomes	Proteasome inhibitor	Multiple myeloma	I/II	NTC00568880
Temsirolimus	Promotes apoptosis and causes the downregulation of mTOR phospho-S6		mTOR	Metastatic solid tumors	I	NTC00909831
Everolimus	Angiogenesis inhibitor; G1 cell-cycle arrest		mTOR	Mantle cell lymphoma	II	NCT00436618
Nab-Paclitaxel (AB-007)	Promotes microtubule assembly and stabilization	Albumin	Cytoskeleton	Breast cancer	Approved	NCT02555696
Rapamycin	Cell viability reduction and decreased mTOR downstream signaling	ABI-007	mTOR	Advanced solid tumors	Ib	NCT00408655, NCT01369433, NCT01014351
Nab-rapamycin (ABI-009)	Cell viability reduction and decreased mTOR downstream signaling	Nanoparticle’s albumin–bound	mTOR targets S6K and 4EBP1	Unresectable advanced non-hematologic malignancies	I	NCT00635284, NCT02646319, NCT03817515
Carboplatin	Cross-linking/alkylating agent	Nab-paclitaxel (ABI-007)	DNA	Advanced or metastatic non–small cell lung cancer	III	NCT02799862
Gemcitabine	Cross-linking/alkylating agent	(ABI-007)	DNA	Metastatic pancreatic adenocarcinoma	II	NCT03529175
		ABI-009 complex		NMIBC	I/II	NTC02009332
3-MA + Trastuzumab (Tmab)	Prevents autophagosome formation	Gold nanoparticle	PI3K inhibition	HER-2 + breast cancer	III	NCT03529110
HCQ + FOLFOX/bevacizumab	Binds and inactivates serum VEGF	ABI-009 complex	Angiogenesis inhibitor	Colorectal cancer	I/II	NCT01206530
HCQ + pazopanib	Antineoplastic agent	ABI-009 complex	VEGF receptor	Sarcoma	I/II	NCT03660930
HCQ + nivolumab	PD-1 blocking antibody	ABI-009 complex	PD-1	Sarcoma, colorectal cancer	I/II	NCT03190174
MRX34	Inhibition of tumor associated EMT	Lipid nanoparticle	MiR-34 specific targets	Solid tumors refractory to standard treatment	I	NCT01829971
SNAs	Targets the oncoprotein Bcl2Like12 a caspase effector and p53 inhibitor overexpressed in GBM	Gold nanoparticle	Bcl2L12 mRNA	Glioblastoma multiforme (GBM)	I	NTC03020017
ND-L02-s0201	Targets heat shock protein 47 (HSP47) in hepatic stellate cells which have key roles in liver homeostasis as well as uptake and storage of vitamin A	Vitamin-A coupled liposome	Heat shock protein 47 (HSP47)	Pulmonary/liver fibrosis	I/II	NCT03538301 NCT03241264 NCT02227459 NCT01858935

**Table 3:** List of preclinical studies and clinical trials testing the efficacy of magnetic nanoparticles in targeting solid tumors

Therapeutic name	Mechanism of action	Delivery system	Target(s)	Cancer type	Phase	NCT ID
MNPs-DOX	Antimitotic and cytotoxic activity	mNPs + pseudopeptide NucAnt(N6L)	DNA of tumor cells	Breast cancer (mice)	preclinical	–
MNPs-GEM	Nucleoside metabolic inhibitor	mNPs	DNA of tumor cells	Pancreatic cancer (PANC-1 cells)	preclinical	–
NanoTherm^®^therapy	Magnetic field hyperthermia in combination with gemcitabine and nab-paclitaxel	mNPs + Albumin	DNA of tumor cells	Prostate	I	NTC02033447
NanoTherm^®^therapy	Magnetic field hyperthermia in combination with gemcitabine and nab-paclitaxel	mNPs + Albumin	DNA of tumor cells	Glioblastoma	I	DKRS00005476

Another phase I/II study (NCT02009332) is taking place to determine the efficacy and dose limiting toxicities (DLT) of ABI-009 nanoparticles combined with gemcitabine as innovative therapy for non-muscle invasive bladder cancer (NMIBC) patients [92]. Intravesical administration of nanoparticles may increase delivery of autophagy inducers across the urothelium, thus potentiating the effect of standard chemotherapeutic gemcitabine, and therefore this study has great potential for improving therapy response of NMIBC. Nab-paclitaxel, (also known as Abraxane or ABI-007), is a Food and Drug Administration–approved treatment for advanced breast cancer [93]. Notably, this nanoformulation has been combined with gemcitabine for metastatic pancreatic cancer, and with carboplatin for locally advanced or metastatic non–small cell lung cancer, resulting in significant antitumor activity in patients [94, 95].

Interestingly, the combination of ABI-007 with rapamycin has been investigated in several phase Ib clinical studies (NCT00408655, NCT01369433, NCT01014351) [96–98]. The tumor of patients treated with weekly doses of rapamycin combined to nab-paclitaxel showed a strong reduction of (^18^F) fludeoxyglucose (FDG) activity which was linked to increased treatment response or stable disease [99]. The 40 mg maximum tolerated dose combined to weekly nab-paclitaxel at 100 mg/m^2^ did not display any toxicity, and the pharmacokinetics of rapamycin showed a coherent relation between dose and plasma concentration, with no significant molecular interplay between rapamycin and nab-paclitaxel detected [99].

## Experimental and clinical potential of nucleic acids in modulating autophagy

The discovery and characterization of non-coding RNA species in the last decades have revolutioned our understanding of genome regulation, and several strategies have aim at leveraging on such molecules for cancer therapy [100]. Among the most largely studied are small interfering RNA (siRNA), antisense oligonucleotides (ASOs), aptamers, micro-RNAs (miRNAs), and plasmid DNA (pDNA) [100, 101].

These molecules display unique proprieties such as good affinity for the target, no immunogenicity and ease of chemical modification, that render them excellent therapeutic systems, particularly to overcome challenges presented by traditional drugs [100]. Their mechanism of action is dependent on the structure and properties of the molecule. For example, siRNA leads RNA-induced silencing complex (RISC) which in turn hampers the mRNA translation of target gene [102], whereas ASOs are able to act either by counteracting the ribonucleoprotein activity or by stimulating the intracellular signaling which lead to mRNA degradation [103].

Autophagy-related miRNAs represent an essential control mechanism on top of all other autophagy-regulatory pathways that were characterized so far. Recently, we have witnessed a drastic increase in the number of studies dissecting miRNA-autophagy relationships [104]. Interestingly, miRNA-34a has been shown to be effective on autophagy-related genes, such as ATG4 [105], ATG5 [106] and ATG9 [107] and has caught remarkable appeal for anticancer treatment. Hence, the rescue of miRNA-34a physiological levels is considered a hopeful opportunity to inhibit cancer progression [108]. For this purpose, gold nanomaterials have been developed to deliver miRNA-34a in different cellular models. The nanoformulation was able to lead to autophagy inhibition, reprogramming cancer cell metabolism and reduce cell proliferation in breast and uveal melanoma cancer cells [109]. In other studies, polymeric nanocomplexes and S6 aptamer-conjugated dendrimers were able to improve the cellular uptake of miRNA-34a in gastric and lung carcinoma cells which targeted the pro-autophagic Notch-1 signaling pathway as well as key genes taking place in autophagy regulation as B-cell lymphoma 2 protein family (Bcl-2) and p53 [110, 111].

Innovative strategies for cancer therapy based on nucleic acids for autophagy modulation have been evaluated in different in vitro and in vivo models as well as have been proposed in clinical studies [112–118]. However, these molecules display certain features such as fast biodegradation, brief half-life in blood flow, low affinity within biological environments, low membrane penetrability, and uncontrolled off-target storage, which have reduced their employment in in vivo systems so far [119]. Considerable effort has thus been focused on improving the targeted delivery of these molecules. Combination with nanostructures may overcome such limitations and generate functional nanomedicines. Different studies employed nanocarriers to deliver autophagy-related nucleic acids, alone or combined to other anticancer agents, in cellular or animal models.

Superparamagnetic iron oxide (Fe_2_O_3_) nanoparticles (SPIONs) have been linked to anti-HER2 antibody and autophagy inhibitor miRNA-376B. These modified nanostructures efficiently delivered the bioactive microRNA into HER2-positive breast tumor cell lines and in a xenograft nude mice model of breast cancer, and lead to autophagy inhibition [120]. Moreover, miRNA-376B-loaded SPIONs drastically increased the anticancer treatment both in vitro in cells and in vivo when combined to the chemotherapy agent cisplatin.

Strategies based on the delivery of siRNAs targeting key genes involved in autophagy regulation also have been successfully exploited for cancer therapy. For example, PEGylated PLGA nanoconjugates decorated with GalNac (GalNAc@PEG@siRNA-PLGA) were designed to carry siRNA targeting survivin, an autophagy regulator gene that has been observed to promote the accumulation and stabilization of IKKα in the nucleus as well as interact with the pro-autophagy functional complex ATG12-ATG5 [121, 122]. The nanoformulation increased the cellular uptake of survivin siRNA as well as its circulation time in animal model, and reduced uptake of siRNA by reticuloendothelial system. This nanomedicine was finally able to lead apoptosis in liver cancer cells and improved survival in HCC-bearing mice [123]. In a related study, to beat multi drug resistance (MDR) in breast cancer cells, cationic nanostructured lipid carriers (NLC) were loaded with doxorubicin (Dox) and ATG7 siRNA to engineer a nanosystem (NLC/D-R) able to downregulate protective autophagy and increase chemotherapy efficacy in breast cancer [124].

In summary, many types of nanomaterials may be modified with specific nucleic acids and drugs to act as nanocarriers to interfere with the complex autophagy process or the related signaling. Therefore, these nanoformulations could be employed for the inhibition of protective autophagy or for the activation of autophagic cell death in combination to chemotherapy injuries and thus leading to a tumor suppressor phenotype which, enhances therapeutic efficacy. Given the potential of gene therapy in modulating autophagy, many clinical studies (NCT03538301, NCT03241264 NCT02227459, NCT01858935, NCT03020017, NCT01829971) have been performed to evaluate the clinical significance of modified nanocarriers in delivering therapeutic nucleic acids able to modulate autophagy process in targeted tissues [125–130]. A list of selected trials involving nanocarriers and nucleic acids is reported here [114].

The high potential of encapsulated miRNAs for clinical use is supported by of MRX34, a lipid nanoparticle loaded with miR-34 mimics, the first microRNA-associated therapeutic molecule tested in a clinical trial [131]. Indeed, a phase I clinical trial on adult patients afflicted by solid tumors resistant to standard therapeutic treatments, showed that a biweekly treatment for 3 weeks with MRX34 exerts a significant antitumoral activity [132]. Interestingly, MRX34 was also found in various tissues, namely liver, bone marrow, spleen, mammary gland, and lung [133], thus supporting its clinical use against various cancer types.

Kasinski et al. showed the therapeutic efficacy of delivered miRNA by using the co-encapsulated miR-34a and let-7b in NSCLC mice resistant to conventional anticancer therapy. Results showed that dual treated animals had lower tumor burden and higher survival [134]. These data warrant further study of encapsulated miRNAs in clinical trials. In a preclinical study spherical nucleic acid (SNAs) nanoparticle conjugates have been developed to efficiently target the oncoprotein Bcl2L12 [135], which is an effector of caspases and has been shown to regulate temozolomide-induced autophagy in glioblastoma multiforme (GBM) [136]. The nanostructures consisted of AuNPs covalently functionalized with small interfering RNA duplexes, and were able to reduce Bcl2L12 expression in an intracerebral GBM model, increase intratumoral apoptosis, as well as reduce tumor progression without side effects [135]. Therefore, counteracting antiapoptotic and pro-autophagic approach using SNAs may be a new strategy against GBM consisting of a systemic RNAi therapy. In this regard, a phase I study (NCT03020017) conducted on 8 patients have not shown treatment related toxicities and showed initial evidence of crossing blood brain [129].

Liver cirrhosis or fibrosis, as the endpoint of chronic hepatic damage, is a strong indicator of high risk for the development of hepatocellular carcinoma (HCC), the main source of cancer-related deaths [137]. Autophagy plays a key role to balance the liver physiology and metabolism. The activation of autophagy has been suggested to avoid liver-associated diseases through autophagic degradation of aggregate-prone proteins and damaged organelles. Thus, a strategy to ameliorate the development of liver disease could be the enhancement of autophagy basal activity [138]. In contrast, autophagy has also been suggested to promote liver damage-induced cell death and the development of liver diseases, which suggests modulation of autophagy may represent a new approach to mitigate the progression of liver diseases. Thus, the physiological importance of autophagy in liver diseases is still debated and highly contextual, and discrepancies among studies warrant further research [138].

Heat shock protein 47 (HSP47) is a molecular chaperone required for collagen folding and maturation. Moreover, HSP47 plays an important role in collagen accumulation in fibrotic areas and disorders associated with desmoplasia [139]. Intriguingly, several reports linked HSP47 with autophagy dysregulation and the development of liver diseases and its knock-down has been explored as a therapeutic approach for various fibroses, including liver cirrhosis [140–142].

A promising preclinical study has developed vitamin A–coupled liposomes able to deliver siRNA targeting HSP47 in hepatic stellate (HS) cells, which have key roles in liver homeostasis and vitamin A uptake and storage [143]. In this study the authors observed that these nanoformulation were effective to resolve liver fibrosis and the siRNA dose used in the in vivo experiments (0.75 mg/kg per single injection) was significantly reduced compared to effective doses previously reported in vivo [144, 145]. This may be explained by the preferential delivery of siRNA HSP47 from the vitamin A-coupled liposomes to HS cells [146]. The high efficacy of this approach in both acute and chronic models of liver fibrosis supports its therapeutic potential against human liver cirrhosis and the safety, tolerability and pharmacokinetic profile of this nanoformulation (ND-L02-s0201) in patients has being evaluated in different promising phase I/II clinical trials (NCT03538301, NCT03241264, NCT02227459, NCT01858935) [125–128].

## Magnetic hyperthermia applications and autophagy

Among different available nanocarriers, metallic nanoparticles have high importance due to their inherent reactivity and physicochemical properties, which can be leveraged on for therapeutic purposes. Therapeutic strategies based on hyperthermia [147] consist in locally producing high temperatures to kill tumor cells or sensitize them to the effects of radiation and specific anticancer drugs. Metallic nanoparticles allow the use of a wide variety of techniques (laser, ionizing radiation and microwaves) to induce heat at sites of nanoparticle accumulation [148]. Magnetic hyperthermia (MH) allows for remote heat induction localized in tumor specific area by using magnetic energy losses in magnetic nanoparticles through administration of alternate magnetic field (AMF), which reduces the side effects at the surrounding healthy tissues [149].

Furthermore, various works highlight magnetic hyperthermia as a promising adjuvant strategy to radiation and chemotherapy against cancer [149]. Alternatively, the exposure of metallic nanoparticles to laser radiation near their plasmon-resonant absorption band allows local heating of nanoparticle-labeled cells avoiding effects in surrounding healthy tissues. In the last years, promising strategies to induce the photothermal effect both in vitro and in vivo have been developed, including plasmonic photothermal therapy (PPTT) [150] and red and near-infrared (NIR) laser light irradiations [151]. MNPs can also be functionalized with active compounds such as doxorubicin, gemcitabine and/or nab-paclitaxel [152–154], to achieve a strong synergistic cytotoxic effect, at least in preclinical models of glioblastoma and pancreatic carcinoma. Some of these preparations have reached clinical trial stages and even approval for their marketing: NanoTherm^®^therapy (trade name), the first MNP based therapy for prostate and brain tumors in the world, has been recently evaluated in a clinical setting (NCT02033447 (prostate) [155] and DRKS00005476 (glioblastoma) [156] for MNPs-MH therapy. In 2013, MagForce AG started the post-marketing clinical study in recurrent glioblastoma with NanoTherm^®^ Therapy with an open label, randomized and controlled trial aimed to determine its efficacy and safety alone or in combination with radiotherapy versus radiotherapy alone. In 2019, the Phase 1 clinical study on the focal ablation of intermediate-risk prostate cancer was completed by MagForce USA, Inc. Hence, to synergistically combine MNPs-MH with other therapeutic approaches, namely chemotherapy, radiotherapy, immunotherapy or photothermal/PDT, will allow a further enhancement of the efficacy against tumors. Although researchers have made a remarkable progress, several challenges stand, as detailed in [157].

Interestingly, hyperthermia has been reported to trigger macro-autophagy across many experimental conditions and in most cases, the induction of this pathway increased cell survival and reduced programmed cell death [158]. Mechanistically, hyperthermia may induce protein unfolding and aggregation leading to induction of heat shock response, which is a main determinant of autophagy induction [159] [160]. In this condition, inhibition of autophagy may enhance the killing effect of hyperthermia in tumor therapy. Accordingly, the chemotherapeutic drug doxorubicin and the autophagy inhibitor chloroquine could enhance the efficacy of nanoparticle-mediated hyperthermia leading to cancer cell death, reduction of tumor volume and improved survival in an in vivo murine model [161–163].

## What is (still) not working with nanoparticles? What needs to be improved?

While nanoparticles have a tremendous potential for antitumoral therapy and extensive research is still been undertaken to assess the viability of their use in the clinic, concerns have raised in this field since despite big efforts along decades, results obtained in terms of technology/knowledge transfer have been so far limited [59, 147]. To date, only five nanoformulations have been approved by FDA for therapy of solid tumors. Moreover, the vast majority of approved drugs consist in liposomes and albumin nanoparticles, relying on technology already available for many years. Last, a NIH-founded NCI Centers of Cancer Nanotechnology Excellence (CCNEs) program was discontinued in 2020, raising speculations in the field about reduced interest on this topic [164]. Two main processes are considered to estimate the bioavailability and the efficacy of drug-covered nanoparticles: i) the EPR effect already discussed above and ii) the reduced uptake of nanoformulations by the reticuloendothelial system (RES). The EPR effect, caused by the leaky vasculature next to the tumor, increases drug accumulation in the affected area.

Although the EPR effect has been confirmed by many studies in nanoparticles, it is still difficult to evaluate its real advantage with respect to free drugs in vivo. The problem resides in the fact that most approved anticancer nanomedicines were analyzed by comparing standard care with the combination of nanomedicine and standard care, instead of comparison with free drugs. Since the free drug may be ineffective for cancer patients, clinical trials using a free drug as a control may not be possible due to ethical concerns [165]. Moreover, the EPR effect has been described to be tumor-specific: for instance, greater EPR effect has been observed in sarcoma with respect to epithelial cancers (e.g. breast cancer) and this may impact the increased effect of encapsulated drugs [165].

Regarding the second effect considered, surface modification of nanoparticles (e.g., PEGylation) limiting uptake by the RES—most prominent in liver and spleen parenchyma—, decreases drug clearance, promotes sustained availability of drug-loaded nanoparticles in the blood stream, and reduces tissue-specific toxicity. This may be relevant to reduce dose requirements and intrinsic toxicity issues, as exemplified by liposome formulation Doxil, with reduced accumulation at the myocardium and cardiotoxicity, as compared to free drug [166].

A further criticism on the lack of translation from in vitro*/*in vivo models to the clinic may be due to the fact that established cell lines conventionally used to validate nanoformulations may do not reflect the heterogeneity existing among human individuals. Even during in vivo assays, the use of subcutaneous injection of tumor cells and xenografts may also provide artifactual results with respect to spontaneous tumor model or metastasis studies [167].

Overall, it should be considered that different anticancer drugs have distinct physicochemical, pharmacokinetic, and pharmacodynamic properties, which determine their unique clinical efficacy and safety profiles in human cancer patients. Moreover, due to tissue specificity of many tumors, different nanodelivery platforms should be designed for different drugs focusing the application on tumor specificities. Indeed drug, specificities (solubility, half-life, tissue distribution/penetration) as well as tumor features (i.e., different EPR effect), should be taken into account.

## Concluding remarks

Cancer is still an unstoppable challenge worldwide, so novel diagnostic and treatment strategies are needed. Among different approaches explored by scientists, nanomedicine emerges as a novel alternative, based on its virtually endless variety of nanomaterials potentially suitable for cancer therapeutics. Thus, different scientific disciplines, such as engineering, chemistry, physics, nanotechnology, materials science or medicine, are integrated to achieve precision systems, which also leverage on existing compounds. However, even though standardization, stability and reproducibility are required for this goal, tailored features are mandatory for the successful application of personalized medicine.

Advanced nanoparticles have been revealed as potential smart drug delivery systems to improve the therapeutic effect of current standard drugs and increase patient survival rates. Undoubtedly, there is still a long journey from the nanocarriers design to translation to the pharmaceutical market as viable products. Although thousands of research articles describe great outcomes of drug delivery systems with different nature and properties in multiple in vitro and in vivo cancer models, only a small fraction has successfully reached trials for their use in the clinic. This limited clinical translation of new nanoparticles is mainly due to incomplete therapeutic efficacy and off-target toxicity in vital organs. Nonetheless, results and evidence from previous clinical trials should guide not only the optimization of nanocarrier formulations, but also setting clinical studies considering tumor heterogeneity through the identification of stratified populations, instead of unbiased cancer patient cohorts.

## Data Availability

Not applicable.
