# Correction to: Nanomedicine for autophagy modulation in cancer therapy: a clinical perspective

**DOI:** 10.1186/s13578-023-01057-9

**Published:** 2023-06-09

**Authors:** Tania B. López-Méndez, Miguel Sánchez-Álvarez, Flavia Trionfetti, José L Pedraz, Marco Tripodi, Marco Cordani, Raffaele Strippoli, Juan González-Valdivieso

**Affiliations:** 1 NanoBioCel Group, University of the Basque Country (UPV/EHU), Vitoria-Gasteiz, Spain; 2 Biomedical Research Networking Center in Bioengineering, Biomaterials and Nanomedicine, (CIBER-BBN), Vitoria-Gasteiz, Spain; 3 Area of Cell and Developmental Biology, Centro Nacional de Investigaciones Cardiovasculares (CNIC), Madrid, Spain; 4 Instituto de Investigaciones Biomédicas Alberto Sols (IIB), Madrid, Spain; 5 Department of Molecular Medicine, Sapienza University of Rome, Rome, Italy; 6 National Institute for Infectious Diseases L. Spallanzani IRCCS, Rome, Italy; 7 Department of Biochemistry and Molecular Biology, School of Biology, Complutense University, Madrid, Spain; 8 Instituto de Investigaciones Sanitarias San Carlos (IdISSC), Madrid, Spain; 9 Molecular Imaging Innovations Institute (MI3), Department of Radiology, Weill Cornell Medicine, New York, USA

**Correction to: Cell & Bioscience (2023) 13:44**

10.1186/s13578-023-00986-9

In this article the statement in the Funding information section was missing and should have read as “Work in the laboratories of the authors is funded by grants from the Italian Ministries for Health (Ricerca Corrente) and grant AIRC IG26290 from AIRC (Italian Association for Cancer Research) to M.T., from Education, University and Research (MIUR; 000003_17_MAP_STRIP and FISR 2020-Covid FISR2020IP_03366) to R.S; and from the Spanish Ministerio de Ciencia e Innovación (PID2021-128106NA-I00 and RYC2020-029690) to M.S-A. M.C. was funded by Ramón y Cajal contract from the Spanish Ministry of Science and Innovation, “Agencia Estatal de Investigación” (MCIN/AEI/10.13039/501100011033), and European Union-NextGenerationEU (EU/PRTR), funding reference:_RYC2021-031003-I). M.C. was also funded by Maria Zambrano contract from the Spanish Ministry of Universities, European Union-NextGenerationEU, and Complutense University of Madrid. 

